# Complex small supernumerary marker chromosomes – an update

**DOI:** 10.1186/1755-8166-6-46

**Published:** 2013-10-31

**Authors:** Thomas Liehr, Sanja Cirkovic, Tanja Lalic, Marija Guc-Scekic, Cynthia de Almeida, Jörg Weimer, Ivan Iourov, Maria Isabel Melaragno, Roberta S Guilherme, Eunice-Georgia G Stefanou, Dilek Aktas, Katharina Kreskowski, Elisabeth Klein, Monika Ziegler, Nadezda Kosyakova, Marianne Volleth, Ahmed B Hamid

**Affiliations:** 1 Jena University Hospital, Friedrich Schiller University, Institute of Human Genetics, Kollegiengasse 10, Jena D-07743, Germany; 2 Laboratory for Medical Genetics, Mother and Child Health Care Institute of Serbia "Dr Vukan Cupic", Radoje Dakic str. 6-8, Belgrade 11070, Serbia; 3 University of Belgrade, Faculty of Biology, Belgrade, Serbia; 4 Military Hospital associated with "Universidad de la República (UDELAR)", Montevideo, Uruguay; 5 Department of Gynecology and Obstetrics, UKSH Campus Kiel, Arnold-Heller-Str. 3; House 24, Kiel 24105, Germany; 6 Research Center for Mental Health, RAMS, Moscow, Russia; 7 Institute of Pediatrics and Children Surgery, RF Ministry of Health, Moscow, Russia; 8 Department of Morphology and Genetics, Universidade Federal de São Paulo, Rua Botucatu 740, São Paulo SP, 04023-900, Brazil; 9 Department of Pediatrics, Laboratory of Medical Genetics, University General Hospital of Patras, Rion, Patras 26504, Greece; 10 Hacettepe University School of Medicine, Dept of Medical Genetics, 06100 Sihhiye, Ankara, Turkey; 11 Institut für Humangenetik, Universitätsklinikum, Leipziger Str. 44, Magdeburg 39120, Germany; 12 Institut für Humangenetik, Postfach, Jena D-07740, Germany

**Keywords:** Complex small supernumerary marker chromosomes (sSMC), Genotype-phenotype correlation, Mosaicism, SSMC shape, Emanuel syndrome

## Abstract

**Background:**

Complex small supernumerary marker chromosomes (sSMC) constitute one of the smallest subgroups of sSMC in general. Complex sSMC consist of chromosomal material derived from more than one chromosome; the best known representative of this group is the derivative chromosome 22 {der(22)t(11;22)} or Emanuel syndrome. In 2008 we speculated that complex sSMC could be part of an underestimated entity.

**Results:**

Here, the overall yet reported 412 complex sSMC are summarized. They constitute 8.4% of all yet in detail characterized sSMC cases. The majority of the complex sSMC is contributed by patients suffering from Emanuel syndrome (82%). Besides there are a der(22)t(8;22)(q24.1;q11.1) and a der(13)t(13;18)(q11;p11.21) or der(21)t(18;21)(p11.21;q11.1) = der(13 or 21)t(13 or 21;18) syndrome. The latter two represent another 2.6% and 2.2% of the complex sSMC-cases, respectively. The large majority of complex sSMC has a centric minute shape and derives from an acrocentric chromosome. Nonetheless, complex sSMC can involve material from each chromosomal origin. Most complex sSMC are inherited form a balanced translocation in one parent and are non-mosaic. Interestingly, there are hot spots for the chromosomal breakpoints involved.

**Conclusions:**

Complex sSMC need to be considered in diagnostics, especially in non-mosaic, centric minute shaped sSMC. As yet three complex-sSMC-associated syndromes are identified. As recurrent breakpoints in the complex sSMC were characterized, it is to be expected that more syndromes are identified in this subgroup of sSMC. Overall, complex sSMC emphasize once more the importance of detailed cytogenetic analyses, especially in patients with idiopathic mental retardation.

## Background

Small supernumerary marker chromosomes (sSMC) are structurally abnormal chromosomes that cannot be identified or characterized in detail by banding cytogenetics, are generally about the size of or smaller than a chromosome 20, and molecular cytogenetic techniques are necessary for their comprehensive characterization [1]. It is estimated that there are ~3 million of sSMC carriers in the human population of 7 billion individuals. Fortunately, only in 1/3 of the cases the sSMC is associated with clinical abnormalities [2]. Besides some specific syndromes, i.e. Pallister-Killian {= i(12p), OMIM #601803}, isochromosome 18p {i(18p), OMIM #614290}, cat-eye {i(22p ~ q), OMIM #115470}, idic(15) {no OMIM number} and Emanuel or derivative chromosome 22 {der(22)t(11;22), OMIM #609029} syndromes [2], for the remaining sSMC-cases only first steps towards genotype-phenotype correlations were achieved [2,3].

sSMC can present with different shapes (ring-, centric minute- and inverted duplication-shape), and consist in the majority of the cases of pericentric chromosomal material. Besides, sSMC can be derived from any part of the human chromosomes and form neocentrics [2,4]. If they derived from the chromosomal ends, in most cases they lead to partial tetrasomies [2]; for one of those conditions also an OMIM entry was introduced recently (#614846 - tetrasomy 15q26 syndrome).

One of the smallest subgroup of sSMC is constituted by the so-called complex marker chromosomes [5]. 'Complex’ are such sSMC which consist of chromosomal material derived from more than one chromosome [1]. Thus, besides the aforementioned large group of Emanuel or derivative chromosome 22 {der(22)t(11;22), OMIM #609029} syndrome cases, there was identified a second recurrent complex sSMC in 2010, designated as supernumerary der(22)t(8;22) syndrome {OMIM #613700} [6].

In 2008 we speculated that the then described 22 complex sSMC cases, excluding the der(22)t(11;22) cases, could be part of an underestimated entity [5]. Here the yet reported 412 complex sSMC cases are summarized based on the sSMC database (http://www.fish.uniklinikum-jena.de/sSMC.html, [3]) and analyzed for their chromosomal constitution, breakpoints and special features.

## Results

**Figure 1:**
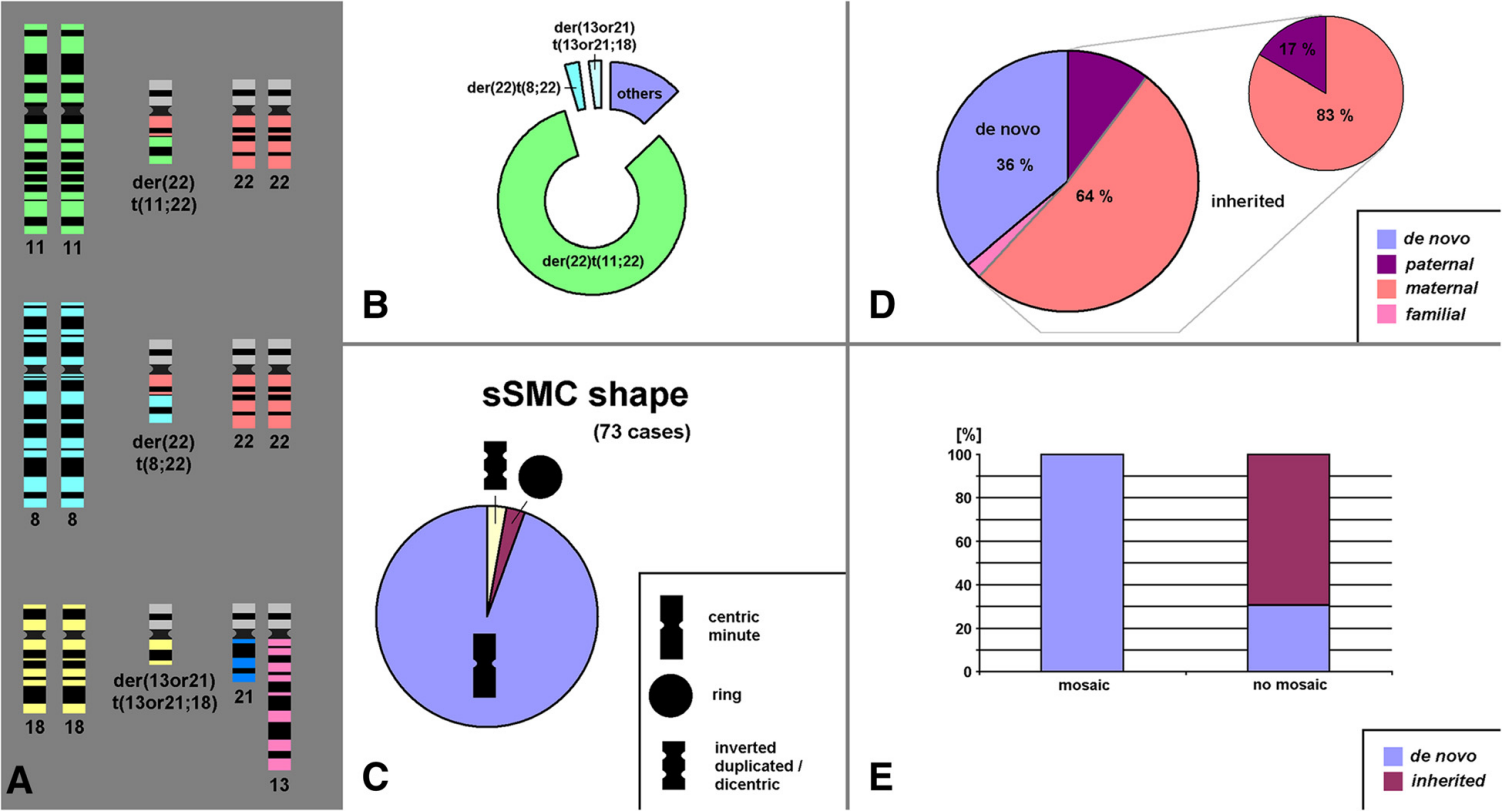
**Complex sSMC: frequencies, shapes, origin and mosaicism. A)** Schematic depictions of the three yet known complex sSMC leading to specific syndromic conditions: the Emanuel = der(22)t(11;22), the der(22)t(8;22) and the der(13 or 21)(13 or 21;18) syndrome. **B)** Frequency of the known three syndromes from **A)** and the other complex sSMC (others) depicted as a ring diagram. **C)** Distribution of the sSMC shapes among the reported complex sSMC cases excluding the cases with Emanuel syndrome. **D)** Distribution of de novo and inherited cases among complex sSMC excluding the cases with Emanuel syndrome. **E)** Complex sSMC tended to be mosaic only among the de novo cases.

The 412 complex sSMC available in literature constitute 8.4% of all yet in detail characterized sSMC cases. The majority of the complex sSMC cases is contributed by der(22)t(11;22)(q23;q11.2) cases, i.e. 339/412 cases (82%). Besides there are two additional types of complex sSMC which have been observed in more than 2 independent patients: the der(22)t(8;22)(q24.1;q11.1) and the der(13)t(13;18)(q11;p11.21) or der(21)t(18;21)(p11.21;q11.1) = der(13 or 21)t(13 or 21;18) (Figure 1A). Both represent another 2.6% and 2.2% of complex sSMC-cases (Figure 1B).

Concerning the shape, complex sSMC present in banding cytogenetics normally as centric minutes: this accounts for all Emanuel syndrome cases and 94% of the remainder ones. Only 2% each of the complex sSMC (excluding Emanuel syndrome cases) occur as inverted duplicated and ring shaped sSMC (Figure 1C). All complex sSMC, apart from one, derive from two chromosomes; only case 07-U-1 is reported to be constituted of three different chromosomes.

As summarized in Table 1, each of the human chromosomes, excluding chromosome 10, was involved in the formation of complex sSMC already. All apart from 14 complex sSMC are derivatives of acrocentric chromosomes. Of the non-acrocentric complex sSMC, derivatives of chromosome 18 were observed most often (3 times).

**Table 1:** Complex sSMC cases summarized from Liehr (2013), not including 339 der(22)t(11;22)(q23;q11.2) cases

**Karyotype**	**Origin**	**Mosaic**	**Gender**	**Case acc. to Liehr (2013)**
der(4)t(4;7)(q12;p22.1)	n.a.	-	F	04-U-10
der(4)t(4;9)(q12;p21.2)	mat	-	F	04-U-11
der(7)t(X;5;7)(p22.1;q35;p13q21)	dn	-	F	07-U-1
der(8;12)(8pter → 8q11.1::12q11.1 → 12pter)	dn	+	M	08-U-10
der(9)t(3;9)(p25;q21.1)	mat	-	F	09-U-22
r(11)t(11;20)(::11p11.1 → 11q12.1::20q13.1?2 → 20q13.32::)	dn	+	F	11-U-12
der(11)t(11;13)(q25;q14)	pat	-	M	11-U-13
der(12)t(4;12)(p16;q11)	mat	-	n.a.	12-U-6
der(13)t(1;13)(q32;q12)	n.a.	-	F	13-U-16
der(13)t(4;13)(q31.3;q13)	mat	-	F	13-U-14
der(13)t(8;13)(p23.1;q12.11)	mat	-	M	13-U-8
der(13 or 21;14)(q10;q10)	n.a.	+	F	13/21-O-q10/4-1
der(13 or 21;15)(q10;q10)	n.a.	-	F	13/21-O-q10/5-1
der(13 or 21)t(13 or 21;18)(q11;p11.2)	dn	-	F	13/21-U-8
der(acro)t(acro;18)(q11;p11.21)	dn	-	F	13/21-U-8d
der(13)t(13;18)(q11;p11.21) or der(21)t(18;21)(p11.21;q11.1)	n.a.	-	F	13/21-U-8a
der(13)t(13;18)(q11;p11.21) or der(21)t(18;21)(p11.21;q11.1)	dn	-	M	13/21-U-8b
der(13)t(13;18)(q11;p11.21) or der(21)t(18;21)(p11.21;q11.1)	n.a.	-	F	13/21-U-8c
der(13)t(13;18)(q11;p11.21) or der(21)t(18;21)(p11.21;q11.1)	n.a.	-	M	13/21-U-8e
der(13)t(13;18)(q11;p11.21) or der(21)t(18;21)(p11.21;q11.1)	dn	-	F	13/21-U-8f
der(13)t(13;18)(q11;p11.21) or der(21)t(18;21)(p11.21;q11.1)	dn	-	M	13/21-U-8g
der(13)t(13;18)(q11;p11.21) or der(21)t(18;21)(p11.21;q11.1)	dn	-	M	+21-U-35
der(14)t(3;14)	mat	n.a.	n.a.	14-U-11
der(14)t(3;14)(p24.1;q21.1)	mat	-	M	14-U-23
der(14)t(5;14)(q13;p13.3)	n.a.	-	F	14-U-12
der(14)t(8;14)(p23;q22)	n.a.	-	M	14-U-27
dic(14;15)(14pter- > 14q11.2::15q11.1- > 15pter)	dn	-	M	14-O-q11.2/1-1
der(14)t(14;16)(q12;q21)	n.a.	-	F	14-U-17
der(14)t(14;17)(q11.2;q25.3)	mat	-	M	14-U-18
der(14)t(14;19)(14pter → 14q11.1::19p13.12 → 19p13.2:)	dn	+	F	14-U-26
der(14 or 22)t(2;14 or 22)(p11.2;q11.1)	dn	+	F	14/22-U-19
der(15)t(15;?)(q24;?)	dn	-	F	15-CW-3
der(15)t(9;15)(p24;q11.2)	mat	-	M	15-O-q11.2/5-1
dic(15;22)(15q11.1;22q22.1)	dn	-	M	15-U-6
der(Y;15)	n.a.	-	F	15-CO-1
der(15)t(Y;15)(q12;q22)	dn	-	M	15-U-10
der(15)t(8;15)(p23.2;q21.3)	dn	-	M	15-U-208
der(15)t(9;15)(p12;q14)	mat	-	F	15-U-189
mar(15;16)	n.a.	n.a.	n.a.	15-U-160
der(15)t(15;16)(q13;p13.2)	mat	-	F	15-U-15
inv dup(13;15)(p11.2p11.2)	n.a.	+	F	15-U-161
der(15)t(15;16)(q13;q13)	mat	-	M	15-U-206
der(15)t(15;16)(q13;p13.2)	mat	-	F	15-U-207
der(15)t(15;17)(q12;q25.3)	mat	-	M	15-U-214
der(15)t(15;18)(q11.1;p11.1 ~ 11.21)	n.a.	-	M	15-U-205
der(17)t(17;acro)(q11;p11.2)	dn	-	M	17-W-p13.3/1-1
der(18)t(2;18)(p23.1;q11.1)	dn	+	F	18-U-24
der(18)t(8;18)(p23.2 ~ 23.1;q11.1)	n.a.	-	M	18-U-10
der(19)t(18;19)	n.a.	n.a.	F	19-U-15
der(18)t(18;21 or 22)	fam	n.a.	n.a.	18-CW-2
der(21)t(4;21)(q32.1;q21.2)	mat	-	F	21-U-15
der(21)t(7;21)(p21;q21.3)	mat	-	M	21-U-7
der(13/21;22)(13/21pter → 13/21q11::22q11.1 ~ 11.2 → 22q11.21 ~ 11.22: :22q11.21 ~ 11.22 → 22pter)	dn	-	F	22-Wces-5-101
der(22)t(6;22)(p22.1;q11.21)	?pat	-	F	22-U-53
der(22)t(8;22)(q24.1;q11.2)	pat	-	M	22-U-11
der(22)t(8;22)(q24.1;q11.1)	mat	-	M/F	22-U-11a1/a2
der(22)t(8;22)(q24.1;q11.1)	pat	-	M	22-U-11b
der(22)t(8;22)(q24.1;q11.1)	mat	-	M	22-U-11c
der(22)t(8;22)(q24.1;q11.1)	mat	-	M	22-U-11d
der(22)t(8;22)(q24.1;q11.1)	mat	-	M	22-U-11e
der(22)t(8;22)(q24.1;q11.1)	mat	-	M	22-U-11f
der(22)t(8;22)(q24.13;q11.21)	n.a.	-	M	22-U-11g
der(22)t(8;22)(q24.13;q11.21)	pat	-	F	22-U-11h
der(22)t(8;22)(q24.1;q11.2)	mat	-	M	22-U-11i
der(22)t(8;22)(q24.1;q11.2)	n.a.	-	M	22-U-11j
der(22)t(8;22)(p22;q11.21)	mat	-	M	22-U-43
der(22)t(9;22)(p13.1;q11)	mat	-	M	22-U-57
der(22)t(12;22)(p12;q11.2-12)	dn	-	M	22-U-18
der(22)t(12;22)(p13.3;q12)	mat	-	M	22-U-18a
der(22)t(14;22)(q31;q11)	mat	-	F	22-O-q11/3-1
der(22)t(17;22)(17pter → 17p10::22q10 → 22pter)	mat	-	M	22-U-6
der(22)t(17;22)(p13.3;q11.21)	pat	-	M	22-O-q11.21/3-1
der(22)t(19;22)(q13.42;q11.1)	n.a.	-	M	22-U-50
r(15)ins(15;5)(?;q35.5q35.3)der(18)(:p11.21 → q11.1:)der(18)(:p11.1 → q11.1:)	dn	+	M	mult 3-9

For 57 of the 73 complex sSMC (excluding Emanuel syndrome) parental studies were done. As depicted in Figure 1D 36% of those were de novo, the remainder ones were inherited form a balanced translocation in one parent. The majority of the latter group (83%) was maternally derived. Interestingly, mosaic cases with karyotypes 47,XN,+mar/46,XN were only seen in de novo complex sSMC (Figure 1E). However, no balanced translocation t(13;18)(q11;p11.21) or t(18;21)(p11.21;q11.1) was seen yet in any of the corresponding nine cases.

In the 73 complex sSMC only 67 breakpoints were involved. 44/67 breakpoints were unique, the remainder observed two to 14 times (Table 2).

**Table 2:** Breakpoints present between two and fourteen times in 73 complex sSMC

**Present X times**	**Breakpoint**
	4q12
	4q31.3 ~ q32.1
	12q11
	13q11 ~ q11.2
	13q13 ~ q14
	14/22q10 ~ q11.1
	15q11.2 ~ 12
	15q21.3 ~ q22
	16p13.2
	17p10 ~ q11
	17q25.3
2	21q21.2 ~ 21.3
	5q35
	14q11.1 ~ 11.2
	15q13
3	22q11.21 ~ q12
4	15q10 ~ q11.1
5	8p22 ~ p23
9	22q11.1 ~ 11.21
10	22q10 ~ 22q11.1
11	8q24.1
	13/21q11
14	18p11.1 ~ 11.21

Finally, only seven of the 73 (~10%) complex sSMC-cases not leading to Emanuel syndrome (case numbers 13/21-O-q10/4-1, 13/21-O-q10/5-1, 14-O-q11.2/1-1, 15-O-q11.2/5-1, 15-CO-1, 22-O-q11/3-1, 22-O-q11.21/3-1) were not associated with clinical signs (Table 1). However, clinically affected carriers of a der(13 or 21)t(13 or 21;18) inherited the sSMC in parts by their mothers, which were considered to be clinically normal [3].

## Discussion

In 2008 complex sSMC seamed to be something rather unusual, apart from the cases with Emanuel syndrome [5]. Since then ~4 times more complex sSMC were characterized and reported, thus enabling more detailed follow up analyses of our previous studies.

~40% (408 of 1,040) of all centric minute shaped sSMC are complex sSMC, including der(22)t(11;22) cases [3]; the latter needs to be kept in mind, if a minute shaped sSMC is detected in diagnostics. Moreover, if a centric minute shaped sSMC turns out to be NOR-positive at one end, thus being acrocentric derived, this means that there is a 70% chance that it is a complex sSMC: of the yet known 567 centric minute shaped sSMC 408 are complex [3]. Also, if a centric minute shaped sSMC is present in all cells of the carrier, this might be another hint for a complex sSMC. Centric minute shaped non-complex sSMC are mosaic in ~70% of the cases [3], while complex sSMC are mosaic in only ~10% of the cases. This indicates the importance of cytogenetic analyses, as only this kind of study enables to characterize the sSMC-shape and mosaicism reliably, and gives first hints on the possible complex nature of an sSMC.

In 2010 the der(22)t(8;22)(q24.1;q11.1) syndrome was reported. It was suggested that, like in Emanuel syndrome, a 3:1 meiotic non-disjunction is causative for the occurrence of the corresponding sSMC in the offspring of t(8;22)(q24.1;q11.1) carriers [6]. Besides in the present study it became obvious that there is at least one more syndrome present among the patients with complex sSMC – nine patients with a der(13 or 21)t(13 or 21;18) were reported yet. It is not known yet if it is always de novo or can also be due to a balanced t(13;18)(q11;p11.21) or t(18;21)(p11.21;q11.1) in one of the parents. However, in contrast to most other complex-sSMC associated syndromes symptoms are very variable, even though a complete trisomy 18p is induced [3].

64% of complex sSMC are due to parental balanced translocations, 36% are de novo. This is a much lower rate that seen in sSMC in general, with a de novo rate of 70% [2; 3]. Still, like in other sSMC the majority of them is maternally derived [2].

At present it seems, complex sSMC fall into two major groups: such with unique and such with (more) common breakpoints. The later group comprises at present 23 different breakpoints involved 2 to 14 times in one of the 73 complex sSMC. As reason for this preference several mechanisms are discussed, including palindrome mediated recurrent translocations [6], homologous recombination between olfactory receptor gene clusters [7] or an involvement of fragile sites in the formation of constitutional breakpoints [8].

While the formation of complex sSMC due to a parental balanced translocation is comprehensible, it is unclear how such sSMC are formed de novo. Mosaicism in the germ-cells of one parent may be a possible explanation. Also, as only de novo cases have been seen in mosaic yet (Figure 1E), postzygotic origin of de novo cases has also to be considered.

As complex sSMC comprise in most cases besides centromeric material also chromosomal parts from gene-rich subtelomeric regions, it is not surprising that in the majority of the cases the clinical consequences are adverse. The seven cases with complex sSMC and no clinical signs only comprised genomic regions without dosage-dependant genes or even only heterochromatin.

## Conclusions

In conclusion, complex sSMC are with 8.4% (including Emanuel syndrome cases) or ~1.5% (excluding der(22)t(11;22) cases) an essential part of the reported sSMC cases. Their frequency was really underestimated in 2008. Especially in cases of clinical abnormal patients with a centric minute shaped sSMC present in 100% of the cells a complex sSMC should be considered.

## Methods

Data was acquired from the freely available sSMC database (http://www.fish.uniklinikum-jena.de/sSMC.html, [3]). 412 sSMC cases were identified as being complex among the 4,913 sSMC cases summarized there. The 339 der(22)t(11;22)(q23;q11.2) cases were not further analyzed; in Table 1 only the details on chromosomal constitution, parental origin, mosaicism and gender for the remainder 73 complex sSMC cases are summarized. Data from Table 1 together with previous knowledge on non-complex sSMC are bases for the here reported and discussed results.

## Competing interests

The authors declare that they have no competing interests.

## Authors’ contributions

SC, TaL, MG-S, CdA, JW, II, IMM, RSG, E-GGS, DA provided the case and/or did primary cytogenetic and parts of FISH-tests; KK, EK, MZ, NK, ABH and TL did detailed FISH studies. TL drafted the paper and all authors read and approved the final manuscript.
